# Trends and Drivers of Inpatient Antibiotic Consumption among 89 China Tertiary General Hospitals from 2011Q1 to 2015Q4

**DOI:** 10.1155/2018/5968653

**Published:** 2018-11-01

**Authors:** Jiangyun Chen, Rui Min, He Wang, Shengwen Zhao, Haomiao Li, Pengqian Fang

**Affiliations:** ^1^School of Medicine and Health Management, Tongji Medical College, Huazhong University of Science and Technology, No. 13 Hangkong Road, Qiaokou District, Wuhan, Hubei 430030, China; ^2^Academy of Health Policy and Management, Huazhong University of Science and Technology, No. 13 Hangkong Road, Qiaokou District, Wuhan, Hubei 430030, China

## Abstract

Antibacterial surveillance is an essential measure for strengthening the management of clinical antibiotic use. This study aimed to determine the trends and drivers of inpatient antibiotic consumption in China. A sample of 89 hospitals with complete data from 2011Q1 to 2015Q4 was included. Accumulative defined daily doses (DDDs), antibiotic use density (AUD), and drug variety were calculated to evaluate antibiotic consumption. From 2011Q1 to 2015Q4, the median values of DDDs, AUD, and drug variety dropped by 10.49%, 39.19%, and 27.96%, respectively. Panel regression results showed, for each additional quarter, DDDs reduced by 6.714 DDDs, AUD reduced by 0.013 DDDs per 100 inpatients per day, and drug variety reduced by 0.012 types (p < 0.001). National hospitals were more likely to use antibiotics, with the highest number of DDDs (106 709 DDDs) and AUD (60 DDDs per 100 inpatients per day) and a large number of drug variety (71 types of drug) all reported from national hospitals. Overall, a downward trend of inpatient antibiotic consumption was observed in competitive tertiary general hospitals in China. However, antibiotic use in China, especially in national hospitals, continues to exceed the guidelines set forth by the nationwide antibiotic stewardship program. China must continue to improve surveillance of antibiotic consumption by constructing a more comprehensive, continuous, and targeted stewardship program. Policy interventions in China should be made in consideration of unbalanced regional development and the consequences this may have on antibiotic consumption.

## 1. Introduction

With dwindling numbers of new antibiotics being developed and widespread inappropriate use of current antibiotics, antibiotic resistance has emerged as a common and growing issue in healthcare institutions worldwide [1]. Excessive use and misuse of antibiotics results in drug resistance and the emergence of “superbugs” [2], increasing the risk of adverse drug reactions [3], potential infections, and even death [4]. Due to challenges in their management and treatment, antibiotic-resistant infections place a heavy burden on the healthcare systems of developing countries [1, 5]. Appropriate antibiotic use and effective stewardship are crucial for global countries in fighting increasing antimicrobial resistance and preventing the worst-case scenario of having “no antibiotic to use” [6–9].

China has a high rate of antibiotic usage for clinical therapy, with an average prescription rate of 72% for antibiotics conditional on prescription [10]. Among Chinese inpatients, 70 out of 100 are using antibiotics [11], which is a significant problem: the maximum rate of inpatient antibiotic usage set by the World Health Organization (WHO) is 30%. Between 2000 and 2015, antibiotic consumption in China increased at a growth rate of 82.6%, from 2.3 to 4.2 billion accumulative defined daily doses (DDDs) [12]. Additionally, as antibiotic prophylaxis effectively prevents postoperative wound infections, antibiotics are widely used in surgery [13]. However, slack surveillance and inappropriate prescription practices in China lead to an average antibiotic resistance growth rate of 22% from 1994 to 2000 [4], which is the fastest growth rate in the world. Considering this evolving public health threat, which imposes an ever-increasing health and economic burden on China, significant clinical and cultural changes are needed.

In other countries, surveillance has played a key role in curbing the overuse of antimicrobials [14–16]. The Chinese government has released several sets of guidelines to improve the rational use of antibiotics, with the first set of guidelines released in 2002. However, initial efforts to improve antibiotic use in China have not been successful. Based on foreign experiences, multifaceted interventions are generally more successful than single strategies [17]. To this end, the Chinese Ministry of Health started a nationwide antibiotic stewardship program (NASP) toward the end of 2011 and formed a special task force to overhaul the clinical use of antibiotics nationwide. This ruling defines all aspects of antibiotic use in Chinese hospitals, including selection, procurement, prescription, use, monitoring, and legal responsibility. Additionally, the NASP stated that no more than 50 kinds of antibacterial drugs should be available in tertiary general hospitals and that the antibiotic use density (AUD) should be below 40 DDDs per 100 patients per day in general hospitals.

It is widely known that antibiotic use and antibiotic resistance patterns are correlated [18–20] and surveillance data on country-level antibiotic use are needed to support policies that aim to reduce antibiotic resistance. Although several studies have attempted to assess antibiotic use in China [21–24], none has reported quarterly data and the influencing factors of inpatient antibiotic consumption. Therefore, this study aimed to determine the trends and influencing factors of Chinese inpatient antibiotic consumption over a more detailed observation time and assess differences according to region and hospital ownership.

## 2. Methods

### 2.1. Data Source

Data used in this study were obtained from the database of the Center for Antibacterial Surveillance (CAS) under the National Health Commission of the People's Republic of China (PRC). CAS currently has 192 core member institutions from all provinces in mainland China (excluding Hong Kong SAR, Macao SAR, and Taiwan province), and all member institutions are public tertiary hospitals (including 181 general hospitals and 11 specialized hospitals). The CAS core member institutions are typically hospitals with relatively high levels of competitiveness in China; of 192 core member institutions, 77 were among China's top 100 competitive hospitals [25].

On a quarterly basis, CAS core member institutions upload inpatient antibiotic consumption data to the CAS according to a unified standard that includes eleven variables: hospital name, province, ownership, time, drug type, drug generic name, dosage form, drug specification, drug amount, inpatient day, and drug cost (Additional file 1).

To ensure data integrity, only CAS core member institutions with complete data were included in this study. Thus, 89 CAS core member institutions who uploaded complete data for each quarter from 2011Q1 to 2015Q4 were selected as the sample hospitals for this study. These hospitals were distributed across 96.8% of the provinces in mainland China. Different regions where the hospitals are located reflect different levels of economic development, and different ownership reflects different levels of competitiveness among the included hospitals. There was no statistical difference in the distribution of region and ownership between the 89 sample hospitals and all 192 CAS core member institutions (Additional file 2). Therefore, the hospitals selected for the study sample were accurate representations of all CAS core member institutions.

Additional information regarding tertiary general hospitals nationwide and basic medical insurance policies in 2011 and 2015 was collected from the National Statistical Yearbook issued by the National Statistical Bureau and the Annual Report on China's Social Insurance Development issued by the Ministry of Human Resources and Social Security of the PRC (Additional file 3).

### 2.2. Definitions of Indicators

For this study, inpatient antibiotic consumption was defined in two ways: as the extent or the intensity of exposure to antibiotics in hospitalized populations. These were measured by three indicators: DDDs, AUD, and drug variety. Each indicator was calculated in a single statistical quarter of each hospital. Larger values of DDDs and drug variety corresponded to higher inpatient antibiotic consumption, whereas a high AUD indicated a higher density of antibiotic consumption per 100 patients per days. The Anatomical Therapeutic Chemical System promoted by the WHO was used as the standard method to measure and report antibiotic use in this study. Classification with defined daily dose (DDD) as the measurement unit was also applied [26]. The formulas used to determine each indicator are as follows:

(1) DDDs=∑drug  specification×amountdefined  daily  doseAUD=DDDs×100inpatient  daysDrug  variety=total  number  of  drug  types

### 2.3. Statistical Analysis

Independent-sample Kruskal–Wallis test was performed to compare the inpatient antibiotic consumption by considering the categorical variables. Estimated generalized least squares regression analysis was performed to assess the effect of time on hospital inpatient antibiotic consumption. DDDs, AUD, and drug variety were taken as the dependent variables. Region, ownership, and time were taken as the independent categorical variables for each regression analysis. Excel and SPSS 22.0 for Windows (IBM Corp, Armonk, NY, USA) were used for descriptive analysis. EViews 8.0 (IHS Global Inc., Engel, Colorado, USA) was used for multivariate regression model analysis. Significance was determined by a* p* value less than 0.05.

## 3. Results

In the present study, the antibiotic consumption of inpatients in 89 tertiary general hospitals across China was analyzed using data from 2011Q1 to 2015Q4. The total number of inpatient days included in the analysis were 300, 0076, and 556.

### 3.1. Distribution of Antibiotic Consumption

Differences in the distribution of DDDs, AUD, and drug variety between different regions, hospital ownership, and across time were statistically significant (*p* < 0.05) (Table 1). DDDs, AUD, and drug variety were complex in regional distribution. Additionally, the data showed that national hospitals were likely to use more antibiotics, with national hospitals reporting the highest median of DDDs (106 709 DDDs), highest median of AUD (60 DDDs per 100 inpatients per day), and bigger median of drug variety (71 types of drug) (Table 1).

### 3.2. Trends of Antibiotic Consumption

**Figure 1:**
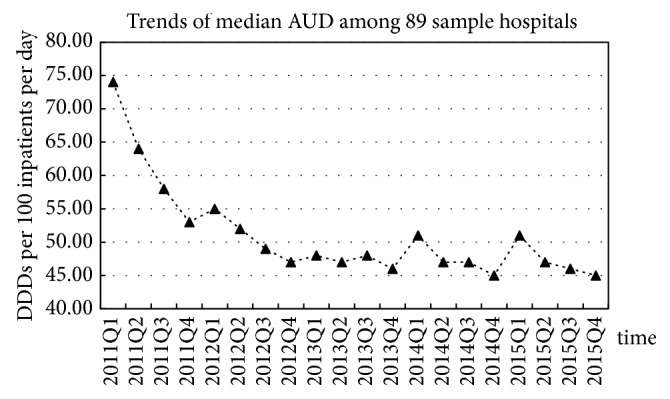
Trends of median AUD among 89 sample hospitals from 2011Q1 to 2015Q4.

From 2011Q1 to 2015Q4, the median values of DDDs, AUD, and drug variety dropped by 10.49% (from 92568 to 82859 DDDs), 39.19% (from 74 to 45 DDDs per 100 inpatients per day), and 27.96% (from 93 to 67 types), respectively (Table 1). The median AUD in the first quarter of each year was often the highest, whereas that in the last quarter was the lowest (Figure 1).

After adjustment for region and ownership, panel regression analysis results showed decreased trends in inpatient antibiotic consumption: for each additional quarter, DDDs reduced by 6.714 DDDs, AUD reduced by 0.013 DDDs per 100 inpatients per day, and drug variety reduced by 0.012 types (p < 0.001; Table 2).

### 3.3. Nationwide Changes in Hospitals and Policies

From 2011 to 2015, the number and capacity of tertiary general hospitals in China increased. The increase in the average number of beds and inpatient days was 10.29% (from 875 to 965 beds) and 4.9% (from 318828 to 334537 inpatient days), respectively. Drug share in China's tertiary general hospitals decreased from 40.30% to 35.47%, and the proportion of central financial subsidies increased from 7.76% to 8.19%. During this period, the basic medical insurance coverage remained around 95%, and the reimbursement policy improved in New Rural Cooperative Medical Insurance, Urban Resident Basic Medical Insurance, and Urban and Rural Resident Basic Medical Insurance (accounting for two-thirds of all basic medical insurance insured persons), as shown in Additional file 3.

## 4. Discussion

Analysis of DDDs, AUD, and drug variety from the five-year data of 89 tertiary general hospitals in China showed a decreasing trend of antibiotic consumption, with the largest decrease in AUD. The most significant decline in AUD was observed in 2011, with a sustained decrease thereafter. The decrease in antibiotic use shown in this study may be closely related to China's recent policy changes and resulting shifts in national conditions.

To guide clinical applications of antibiotic use management, the NASP was proposed in China in 2011 and continuously implemented in the succeeding years. The NASP stipulated that no more than 50 kinds of antibiotics could be used in tertiary general hospitals, with no more than 40 DDDs per 100 inpatients per day. Previous research has shown that antimicrobial combinations should be reduced to avoid antimicrobial resistance [27]. Thus, to some extent, limiting drug variety can reduce the available drug combinations. With a median value of 45 DDDs per 100 inpatients per day and 67 types of antibiotics reported in in the final quarter in this study, it is clear that the requirements for both AUD and drug variety have not been met.

Importantly, this study found that national hospitals were more likely to use antibiotics and were also more likely to have a greater variety of drugs available. National hospitals have the best medical resources and attract higher numbers of inpatients compared to municipal or provincial hospitals [28]. This leads to a higher usage of antibiotics. The per capita consumption of antibiotics in China is higher than that in at least 75% of 29 European countries, in terms of the consumption of third- and fourth-generation cephalosporin and fluoroquinolones [24]. Thus, increased efforts are needed to curb antibiotic use in China, with particular emphasis placed on the supervision of antibiotic use in national hospitals.

Regarding its public hospital reform policy, China launched the reform of public hospitals in 2009, proposed a reconstruction compensation mechanism, and gradually changed the compensation for public hospitals from service charges, drug add-on income, and government subsidies to service charges and government subsidies [4, 29]. In April 2012, the General Office of the State Council issued a notice on “Deepening the Major Work Arrangements for the Reform of the Medical and Health System in 2012,” stating that the reform of public hospitals will cancel drug add-on income (not including traditional Chinese medicine) [30]. As we have seen, drug share in China's tertiary hospitals decreased from 2011 to 2015. Thus, it is possible that removal of drug add-on income might has corresponded to the decline in antibiotic consumption.

In terms of changes in social security and health status in China, the average life expectancy has increased from 74.8 years in 2010 to 76.3 years in 2015 [31], and chronic noninfectious diseases have replaced infectious diseases as the main disease burden [32]. These changes correspond to accelerating industrialization and urbanization, a growing elderly population, and steady improvements in coverage and reimbursement of basic medical insurance policies. According to the “China Cardiovascular Disease Report 2015,” death from cardiovascular disease ranks first among the total deaths of urban (42.51%) and rural (44.6%) residents [33]. Changes in the disease spectrum at the population level, as well as in basic medical insurance coverage and reimbursement, are consistent with the increase in numbers and capacity of tertiary general hospitals in China between 2011 and 2015; to some extent, these changes might also explain the reduction in antibiotic use shown in this study.

In the present study, the AUD was often the highest in the first quarter of each year and lowest in the last quarter. This finding might be due to the medical insurance reimbursement policy known as “global budget”, which provides a hospital with medical insurance funding for one year [34]. At the end of each year, when the budget exceeds the funding received, treatment plans for patients are reduced until the new budget is set and funding received for the next year. Thus, more antibiotics might be used for treatment in the first quarter than in the last quarter. In addition, the Chinese Spring Festival is held in the first quarter of each year. As the number of patients has been shown to increase after the Spring Festival [35], the number of inpatient days could be smaller in the first quarter compared to that in the other quarters.

Inpatient antibiotic consumption in China was also shown to be affected by region. A previous study reported that the increase in global consumption of antibiotics was primarily due to increased antibiotic consumption in low- and middle-income countries (LMICs) [12]. Complex interactions exist between the levels of economic well-being [4], and inequities in drug access persist because many LMICs continue to be burdened with high rates of infectious disease-related mortality and low rates of antibiotic consumption [12]. Therefore, policy interventions in China should be made in consideration of unbalanced regional development and the consequences this may have on antibiotic consumption.

This study has several limitations. First, according to the inclusion criteria and available complete data, only 89 hospitals were selected. The data of these hospitals can indicate trends of antibiotic use in China to some extent but should not be taken as they are not representative for non-CAS core member institutions, especially for hospitals that are less competitive. Future research should be conducted in less competitive hospitals. Second, the database was derived from CAS rather than from hospitals directly. Thus, the variables involved were limited, and it is possible that some important variables were not analyzed. Therefore, some useful variables were extracted from the national public database to help explain the downward trend in antibiotic use. Third, we calculated the cumulative AUD of each hospital rather than the individual AUDs of antibacterial drugs, thereby ignoring some of the increasing tendencies of the AUDs of certain drugs. Fourth, we were unable to determine the appropriateness of antibiotic use at the individual level because the study analyzed the antibiotic consumption data of hospitals rather than that of individual patient prescriptions.

## 5. Conclusion

The results of this study indicate a decreasing trend in inpatient antibiotic consumption in China, which may be closely related to recent changes in national policy and economic conditions. However, antibiotic use in China, especially in national hospitals, continues to exceed the guidelines set forth by the NASP. National policy should take into consideration the effects of unbalanced economic development on healthcare at the regional level. Additionally, as evidenced by the high numbers of DDDs and AUD in national hospitals, supervision of antibiotic use should be strengthened, particularly in national hospitals. Although some progress has been achieved by recent reforms, China must continue to improve surveillance of drug resistance and antimicrobial drug consumption and reduce the unnecessary use of antibiotics by constructing a more comprehensive, continuous, and targeted stewardship program.

## Figures and Tables

**Table 1:** Distribution of antibiotic consumption [n = 1780].

Indicator	Variable	Median	*IQR*	
DDDs	Regions			*H*=177.050 *p* < 0.001
	Eastern	65227	43265	
	Central	88855	95542	
	Western	79697	33997	
	Northeastern	90053	72565	
	Ownership			*H*=94.684 *p* < 0.001
	National hospital	106709	115665	
	Provincial hospital	76162	46415	
	Municipal hospital	68675	45485	
	Time^a^			*H*=38.313 *p*=0.005
	2011Q1	92568	85936	
	2015Q4	82859	60562	

AUD	Regions			*H*=52.759 *p* < 0.001
	Eastern	50	19	
	Central	54	26	
	Western	46	17	
	Northeastern	51	24	
	Ownership			*H*=63.253 *p* < 0.001
	National hospital	60	32	
	Provincial hospital	48	20	
	Municipal hospital	51	19	
	Time^a^			*H*=220.919 *p* < 0.001
	2011Q1	74	33	
	2015Q4	45	17	

Drug variety	Regions			*H*=47.982 *p* < 0.001
	Eastern	71	17	
	Central	72	26	
	Western	69	23	
	Northeastern	65	15	
	Ownership			*H*=24.316 *p* < 0.001
	National hospital	71	20	
	Provincial hospital	67	19	
	Municipal hospital	73	21	
	Time^a^			*H*=247.050 *p* < 0.001
	2011Q1	93	34	
	2015Q4	67	17	

IQR, interquartile-range; DDDs, accumulative defined daily doses; AUD, antibiotic use density.

DDDs = ∑ (drug specification × amount/defined daily dose).

AUD = DDDs × 100/inpatient days, presented as “DDDs per 100 inpatients per day”.

Drugs variety refers to the total number of drug types, presented as “kinds”.

^a^Test for all quarters.

**Table 2:** Panel regression analysis predicting likelihood of the use of antibiotic.

Dependent variable	Variable^a^	Coefficient	*t*	*p*
DDD^b^	Regions	10779.312	8.315	<0.001
	Ownership	−20711.436	−9.714	<0.001
	Time	−6.714	−2.546	0.011
	Constant	5051175.817	2.606	0.009

AUD^c^	Regions	−0.152	−0.272	0.786
	Ownership	−1.596	−1.733	0.083
	Time	−0.013	−11.610	<0.001
	Constant	9978.110	11.682	<0.001

Drug variety^d^	Regions	−1.346	−3.346	0.001
	Ownership	−0.082	−0.125	0.901
	Time	−0.012	−14.526	<0.001
	Constant	8811.157	14.653	<0.001

DDDs, accumulative defined daily doses.

DDDs = ∑ (drug specification × amount/defined daily dose).

AUD = DDDs × 100/inpatient days.

Drugs variety refers to the total number of drug types.

Total panel (balanced) observations: 1780.

^a^Adjusted for all covariates listed. There is no collinearity between variables.

^b^F-Statistic: 62.070, prob. (F-Statistic): <0.001.

^c^F-Statistic: 45.940, prob. (F-Statistic): <0.001.

^d^F-Statistic: 74.080, prob. (F-Statistic): <0.001.

## Data Availability

The data used to support the findings of this study are available from the corresponding author upon request.

## Supplementary Materials

Supplementary Materials Additional file 1: list and description of included data variables. Additional file 1 shows the definition and description of included variables from the database of CAS. Additional file 2: sample hospitals distribution. Additional file 2 shows the distribution and comparison of 89 samples of hospitals and all 192 CAS core member institutions. Additional file 3: characteristics of tertiary general hospitals nationwide and insurance policies in 2011 and 2015. Additional file 3 shows nationwide data of tertiary general hospitals and basic medical insurance policies in 2011 and 2015.

## References

[1] Sharma A. (2011). Antimicrobial resistance: no action today, no cure tomorrow. *Indian Journal of Medical Microbiology*.

[2] Ferber D. (2000). Superbugs on the hoof?. *Science*.

[3] Phelps C. E. (1989). Bug/drug resistance sometimes less is more. *Medical Care*.

[4] Zhang R., Eggleston K., Rotimi V., Zeckhauser R. J. (2006). Antibiotic resistance as a global threat: evidence from China, Kuwait and the United States. *Globalization and Health*.

[5] Meng Q., Xu L., Zhang Y. (2012). Trends in access to health services and financial protection in China between 2003 and 2011: A cross-sectional study. *The Lancet*.

[6] Kmietowicz Z. (2000). WHO warns of threat of "superbugs".. *BMJ (Clinical research ed.)*.

[7] Klugman K., Koornhof H. (1989). Worldwode increase in pneumococcal antibiotic resistance. *The Lancet*.

[8] Kondro W. (2002). Canadian scientists urge government to develop antibiotic plan.. *The Lancet*.

[9] Aldeyab M. A., Kearney M. P., Scott M. G. (2012). An evaluation of the impact of antibiotic stewardship on reducing the use of high-risk antibiotics and its effect on the incidence of Clostridium difficile infection in hospital settings. *Journal of Antimicrobial Chemotherapy*.

[10] Currie J., Lin W., Zhang W. (2011). Patient knowledge and antibiotic abuse: Evidence from an audit study in China. *Journal of Health Economics*.

[11] Hu S., Liu X., Peng Y. (2003). Assessment of antibiotic prescription in hospitalised patients at a Chinese university hospital. *Infection*.

[12] Klein E. Y., Van Boeckel T. P., Martinez E. M. (2018). Global increase and geographic convergence in antibiotic consumption between 2000 and 2015. *Proceedings of the National Acadamy of Sciences of the United States of America*.

[13] Bowater R. J., Stirling S. A., Lilford R. J. (2009). Is antibiotic prophylaxis in surgery a generally effective intervention?: testing a generic hypothesis over a set of meta-analyses. *Annals of Surgery*.

[14] Crouch D. (2003). Tackling the new superbugs.. *Nursing Times*.

[15] Han K. S., Ramsamy Y. (2013). Surveillance alone plays a key role in curbing the overuse of antimicrobials: The major role of antibiotic stewardship. *South African Medical Journal*.

[16] Johnson A. P. (2013). Improving antimicrobial stewardship: AmWeb, a tool for helping microbiologists in England to 'Start Smart' when advising on antibiotic treatment. *Journal of Antimicrobial Chemotherapy*.

[17] Regev-Yochay G., Raz M., Dagan R. (2011). Reduction in antibiotic use following a cluster randomized controlled multifaceted intervention: The Israeli judicious antibiotic prescription study. *Clinical Infectious Diseases*.

[18] Liu S., Wang C., Fu Y. X. (2017). Analysis of drug resistance of Acinetobacter baumannii in wound of children with traffic injury and its relationship with antibiotic use. *Zhonghua shao shang za zhi = Zhonghua shaoshang zazhi = Chinese journal of burns*.

[19] Almagor J., Temkin E., Benenson I., Fallach N., Carmeli Y., Zhou Z. (2018). The impact of antibiotic use on transmission of resistant bacteria in hospitals: Insights from an agent-based model. *PLoS ONE*.

[20] Sahman-Zaimovic M., Vukmirovic S., Tomic N., Stilinovic N., Horvat O., Tomic L. (2017). Relationship between outpatient antibiotic use and the prevalence of bacterial infections in Montenegro. *Vojnosanitetski Pregled*.

[21] Bao L., Peng R., Wang Y. (2015). Significant reduction of antibiotic consumption and patients' costs after an action plan in China, 2010-2014. *PLoS ONE*.

[22] Zhang Q. Q., Ying G. G., Pan C. G., Liu Y. S., Zhao J. L. (2015). Comprehensive evaluation of antibiotics emission and fate in the river basins of China: source analysis, multimedia modeling, and linkage to bacterial resistance. *Environmental Science & Technology*.

[23] Li C., Ren N., Wen X. (2013). Changes in Antimicrobial Use Prevalence in China: Results from Five Point Prevalence Studies. *PLoS ONE*.

[24] Wushouer H., Tian Y., Guan X.-D., Han S., Shi L.-W. (2017). Trends and patterns of antibiotic consumption in China’s tertiary hospitals: Based on a 5 year surveillance with sales records, 2011-2015. *PLoS ONE*.

[25] Asclepius Healthcare Top 100 competitiveness China hospitals in 2016. http://www.ailibi.com/index.php/portal/rank/article/id/126.

[26] Kuster S. P., Ruef C., Bollinger A. K. (2008). Correlation between case mix index and antibiotic use in hospitals. *Journal of Antimicrobial Chemotherapy*.

[27] Master R. N., Clark R. B., Karlowsky J. A., Ramirez J., Bordon J. M. (2011). Analysis of resistance, cross-resistance and antimicrobial combinations for Pseudomonas aeruginosa isolates from 1997 to 2009. *International Journal of Antimicrobial Agents*.

[28] Dan M. (2007). *Performance and scale economy analysis for minisrty's general hospital of China, master [master, thesis]*.

[29] Xinhua News Opinions of the Central Committee of the Communist Party of China on Deepening the Reform of the Medical and Health System. http://www.gov.cn/jrzg/2009-04/06/content_1278721.htm.

[30] Zhu C., Mao Z. (2012). Cancel the medical supplement mechanism to deepen the reform of public hospitals. *Qiu Shi*.

[31] National Bureau of Statistics of China Life expectancy. *National Data*.

[32] Yang T. A., Li B., Sun L. Z. (2011). Advances in research on prevention and control of chronic non-communicable diseases in China. *Journal of Medical Research*.

[33] Chen W. W., Gao R. L., Liu L. S. (2017). China cardiovascular diseases report 2015: a summary. *Journal of Geriatric Cardiology*.

[34] Wang Z. F. (2017). Medical insurance global budget: Questions and Reflections. *China Social Security*.

[35] Li C. F., Ke S. S., Liu X. H., Yan Y. Q., Li F., Wang L. (2017). Seasonal trends of inpatient number and hospital expenses in primary health care facilities. *Chinese Journal of Social Medicine*.

