# Supplementary material for: Trends and Drivers of Inpatient Antibiotic Consumption among 89 China Tertiary General Hospitals from 2011Q1 to 2015Q4
Source: Biomed Res Int. 2018 Nov 1;2018:5968653. doi: 10.1155/2018/5968653 (PMC6241243; doi:10.1155/2018/5968653)
Supplement: Supplementary Materials — Additional file 1: list and description of included data variables. Additional file 1 shows the definition and description of included variables from the database of CAS. Additional file 2: sample hospitals distribution. Additional file 2 shows the distribution and comparison of 89 samples of hospitals and all 192 CAS core member institutions. Additional file 3: characteristics of tertiary general hospitals nationwide and insurance policies in 2011 and 2015. Additional file 3 shows nationwide data of tertiary general hospitals and basic medical insurance policies in 2011 and 2015. [file 5968653.f1.pdf]

Additional file 1: List and description of included data variables.

Variable	Description
Hospital name	Official name of the hospital.
Province	Province where the hospital was located. There are a total of 31 provinces in mainland China (excluding Hong Kong SAR, Macao SAR, and Taiwan province).
Region [a]	The region where the hospital was located. Based on differences in socio-economic development, mainland China is divided into four regions: eastern China, central China, western China, and western China; these include 10, 6, 12 and 3 provinces, respectively.<br>Eastern China has the highest economic development, followed by central China, western China, and northeast China.
Ownership	In terms of property rights, national hospitals belong to the central government, provincial hospitals belong to the local provincial government, and municipal hospitals belong to the local municipal government.<br>In China, national hospitals are the most competitive, followed by provincial hospitals and municipal hospitals.
Time	Quarter in which data was reported, from 2011Q1 to 2015Q4.
Drug type	Anatomical Therapeutic and Chemical (ATC) classification J01.
Drug generic name	A drug marketed under its chemical name without advertising.
Dosage form	Unit dose, referring to the pharmaceutical drug product in the form in which it was marketed for use.
Drug specification	Referring to the weight (or potency), content (%), or loading of the main drug in each tablet or other unit preparation.
Drug amount	Number of antibiotics used.
Inpatient day	Sum of the length of stay for all inpatients during the specified period; = number of hospitalization days for discharged patients × number of admissions.
Drug cost	The money spent on one specified generic drug.

[a] Region was not captured in the database of the Center for Antibacterial Surveillance and was provided by the researchers according to province where the hospital was located.

Addition file 2. Sample hospitals distribution.

Variables	Sample hospitals		CAS member hospitals		Chi-square test	
	n = 89	%	n = 192	%	χ^2	*p*
Province (n = 31)	30	96.77	31	100.00	1.016	0.313
Regions					2.337	0.505
Eastern	38	42.70	93	48.43		
Central	22	24.72	35	18.23		
Western	17	19.10	43	22.40		
Northeastern	12	13.48	21	10.94		
Ownership					0.667	0.716
National hospital	11	12.36	29	15.10		
Provincial hospital	46	51.69	102	53.13		
Municipal hospital	32	35.95	61	31.77		

Abbreviation: CAS, the Center for Antibacterial Surveillance.

Additional file 3: Characteristics of tertiary general hospitals nationwide and insurance policies in 2011 and 2015.

Characteristic	2011	2015
China's tertiary general hospitals [a]		
Number of China's tertiary general hospitals	1399	2123
Proportion of central financial subsidy (%)	7.76	8.19
Drug share (%)	40.30	35.47
Average number of beds (bed)	874.61	964.59
Average hospitalization day for discharged patients (day)	12.00	10.40
Average number of admissions (person)	26569	32167
Average inpatient days (person day)	318828.00	334536.80
Basic medical insurance policy [b]		
Basic medical insurance coverage (%)	94.91	96.51
Total population (billion people)	13.75	13.47
Number of UEBMI participants (billion people)	2.52	2.89
Number of NRCM participants (billion people)	8.32	6.34
Number of URBMI and URRBMI participants (billion people)	2.21	3.77
Actual medical guarantee provided of UEBMI in hospitalization (%)	73.00	72.80
Actual medical guarantee provided of NRCM, URBMI and URRBMI in hospitalization (%)	58.90	60.20

Abbreviation: UEBMI, Urban Employee Basic Medical Insurance; NRCM, New Rural Cooperative Medical Insurance; URBMI, Urban Resident Basic Medical Insurance; URRBMI, Urban and Rural Resident Basic Medical Insurance

Proportion of central financial subsidy = central financial subsidy / total hospital income × 100%

Drug share = drug income / total hospital income × 100%

Inpatient days = number of hospitalization days for discharged patients × number of admissions

Insurance coverage = number of UEBMI, NRCM, URBMI and URRBMI participants / total population × 100%

Actual compensation ratio = aggregate insurance benefit received in a single policy year / total medical expenses × 100%

[a] data was collected from *The National Statistical Yearbook* issued by the National Bureau of Statistical of China [URL:http://navi.cnki.net/KNavi/YearbookDetail?pcode=CYFD&pykm=YSIFE&bh=]

[b] data was collected from *The Annual Report on China's Social Insurance Development* issued by the Ministry of Human Resources and Social Security of China [URL:http://www.mohrss.gov.cn/gkml/shbx/shbxjb/201802/t20180206_288037.html]
